# Hurricane Season Public Health Preparedness, Response, and Recovery Guidance for Health Care Providers, Response and Recovery Workers, and Affected Communities — CDC, 2017

**DOI:** 10.15585/mmwr.mm6637e1

**Published:** 2017-09-22

**Authors:** CDC 2017 Hurricane Incident Management System Team

**Affiliations:** ^1^; 1 Emergency Operations Center, CDC.

CDC and the Agency for Toxic Substances and Disease Registry (ATSDR) have guidance and technical materials available in both English and Spanish to help communities prepare for hurricanes and floods (Table 1). To help protect the health and safety of the public, responders, and clean-up workers during response and recovery operations from hurricanes and floods, CDC and ATSDR have developed public health guidance and other resources; many are available in both English and Spanish (Table 2).

**TABLE 1:** English and Spanish community guidance for preparing for hurricanes and floods — CDC, 2017

English	En Español
Information about hurricanes and other tropical storms https://www.cdc.gov/disasters/hurricanes/index.html	Huracanes y otras tormentas tropicales https://www.cdc.gov/es/disasters/hurricanes/index.html
Preparations before a hurricane https://www.cdc.gov/disasters/hurricanes/before.html	Antes de un huracán https://www.cdc.gov/es/disasters/hurricanes/before.html
Family, health, and safety preparation https://www.cdc.gov/disasters/hurricanes/supplies.html	Obtenga suministros https://www.cdc.gov/es/disasters/hurricanes/supplies.html
Key facts about flood readiness https://www.cdc.gov/disasters/floods/readiness.html	Datos importantes sobre los preparativos para una inundación https://www.cdc.gov/es/disasters/floods/readiness.html

**TABLE 2:** English and Spanish guidance for response and recovery from hurricanes and floods, by primary target audience — CDC, 2017

English	En Español
**General audience**	
Be safe after a hurricane* https://www.cdc.gov/disasters/hurricanes/be-safe-after.html	Manténgase a salvo después de un huracán https://www.cdc.gov/es/disasters/hurricanes/be-safe-after.html
After a hurricane https://www.cdc.gov/disasters/hurricanes/after.html	Después de un huracán https://www.cdc.gov/es/disasters/hurricanes/after.html
Floods (general information) https://www.cdc.gov/disasters/floods/index.html	Información sobre inundaciones https://www.cdc.gov/es/disasters/floods/index.html
After a flood https://www.cdc.gov/disasters/floods/after.html	Después de una inundación https://www.cdc.gov/es/disasters/floods/after.html
Flood waters or standing waters health risks https://www.cdc.gov/healthywater/emergency/extreme-weather/floods-standingwater.html	Agua de la inundación después de un desastre o una emergencia https://www.cdc.gov/es/disasters/floods/cleanupwater.html
Building and facilities damage: health risks https://www.cdc.gov/healthywater/emergency/extreme-weather/building-damage.html	—^†^
Cleaning up your home after a disaster or emergency https://www.cdc.gov/disasters/hurricanes/cleanup-home.html	Limpiar tu casa después de un desastre o emergencia Limpie su casa https://www.cdc.gov/es/disasters/hurricanes/cleanup-home.html
Generator and furnace safety https://www.cdc.gov/co/pdfs/Generators.pdf https://www.cdc.gov/co/pdfs/Furnace.pdf	Seguridad con los Generadores y Calentadores https://www.cdc.gov/co/pdfs/flyers_Spanish.pdf
Pressure washer safety https://www.cdc.gov/disasters/pressurewashersafety.html	—
Carbon monoxide poisoning^§^ https://www.cdc.gov/co/pdfs/Flyer_Danger.pdf	Intoxicación por monóxido de carbono https://www.cdc.gov/co/pdfs/campaign_flyer_ES.pdf
Carbon monoxide poisoning FAQs https://www.cdc.gov/co/faqs.htm	Intoxicación con Monóxido de Carbono Preguntas Frecuente https://www.cdc.gov/co/es/faqs.htm
Chemical hazards: asbestos in your environment: what you can do to limit exposure https://www.atsdr.cdc.gov/docs/limitingenvironmentalexposures_factsheet-508.pdf	—
ToxFAQ for asbestos https://www.atsdr.cdc.gov/toxfaqs/tf.asp?id = 29&tid = 4	ToxFAQs Asbesto (Amianto) https://www.atsdr.cdc.gov/es/toxfaqs/es_tfacts61.html
Chemical hazards: mercury https://www.atsdr.cdc.gov/dontmesswithmercury/index.html	No te metas con mercurio https://www.atsdr.cdc.gov/dontmesswithmercury/es/index.html
Chemical hazards: lead https://www.cdc.gov/nceh/lead/tips.htm	Lo que debe saber sobre el envenenamiento del plomo https://www.cdc.gov/nceh/lead/tools/know_the_factsspanish.pdf
Coping with a disaster or traumatic event https://emergency.cdc.gov/coping/index.asp	Cómo enfrentar un desastre o evento traumático https://emergency.cdc.gov/es/coping/index.asp
Food safety for infants after a disaster https://www.cdc.gov/breastfeeding/recommendations/food-safety-for-infants-after-a-disaster.html	Asegúrese de que los alimentos y el agua se puedan consumir sin correr riesgo (Cómo alimentar a su bebé) https://www.cdc.gov/es/disasters/hurricanes/foodwater.html
Keep food and water safe after a disaster https://www.cdc.gov/disasters/foodwater/facts.html	Asegúrese de que los alimentos y el agua se puedan consumir sin correr riesgo https://www.cdc.gov/es/disasters/hurricanes/foodwater.html
Personal hygiene and handwashing after a disaster or emergency https://www.cdc.gov/disasters/floods/sanitation.html	Higiene personal y lavado de manos después de un desastre o emergencia https://www.cdc.gov/es/disasters/floods/sanitation.html
Extreme heat https://www.cdc.gov/disasters/extremeheat/index.html	Calor Extremo y Su Salud https://www.cdc.gov/extremeheat/espanol/index_esp.html
Homeowner’s and renter’s guide to mold cleanup after disasters https://www.cdc.gov/mold/pdfs/homeowners_and_renters_guide.pdf	Guía del propietario y arrendatario para la limpieza de moho después de desastres https://www.cdc.gov/mold/pdfs/IEPWG_Mold_Homeowners_and_Renters_Spanish_508.pdf
Get rid of mold https://www.cdc.gov/disasters/hurricanes/pdf/flyer-get-rid-of-mold.pdf	Elimine el moho https://www.cdc.gov/es/disasters/hurricanes/pdf/flyer-get-rid-of-mold.pdf
Mold FAQs https://www.cdc.gov/mold/faqs.htm	Preguntas más frecuentes sobre molde https://www.cdc.gov/mold/es/faqs.htm
*Ready Wrigley Prepares for Storm and Flood Recovery* (a resource for children) https://www.cdc.gov/phpr/readywrigley/documents/17_279940_Ready_Wrigley_mold_508.pdf	—
More resources for families https://www.cdc.gov/disasters/hurricanes/more-resources.html	Más recursos para las familias https://www.cdc.gov/es/disasters/hurricanes/more-resources.html
Public service announcements (PSAs) https://www.cdc.gov/disasters/hurricanes/psa.html	Anuncios de servicio público (PSA) https://www.cdc.gov/es/disasters/hurricanes/psa.html
**Health care professionals**	
Medical care of ill disaster evacuees: additional diagnoses to consider https://www.cdc.gov/disasters/medcare.html	—
Medical management and patient advisement after a disaster https://www.cdc.gov/disasters/management.html	—
Clinical guidance for carbon monoxide (CO) poisoning after a disaster https://www.cdc.gov/disasters/co_guidance.html	Directrices clínicas para la intoxicación por monóxido de carbono (CO) después de un desastre https://www.cdc.gov/es/disasters/co_guidance.html
Safety information for health care professionals https://www.cdc.gov/disasters/hurricanes/hcp.html	Información de seguridad para los profesionales de la salud https://www.cdc.gov/es/disasters/hurricanes/hcp.html
**Public health professionals and response workers**	
Emergency: response resources for storm, flood, and hurricane response https://www.cdc.gov/niosh/topics/emres/flood.html	NIOSH advierte sobre los peligros de limpieza después de una inundación https://www.cdc.gov/spanish/NIOSH/docs/94-123_sp/
Death scene investigation after natural disaster or other weather-related events: a toolkit https://www.cdc.gov/nceh/hsb/disaster/docs/DeathSceneInvestigation508.pdf	—
Public health assessment and surveillance after a disaster https://www.cdc.gov/disasters/surveillance/	Formas de vigilancia de mortalidad relacionadas con desastres están disponibles en español https://www.cdc.gov/es/disasters/surveillance/pdf/disaster-mortality-instructions.pdf https://www.cdc.gov/es/disasters/surveillance/pdf/disaster-mortality-form.pdf
Community Assessment for Public Health Emergency Response (CASPER) https://www.cdc.gov/nceh/hsb/disaster/casper/	—
Emergency Responder Health Monitoring and Surveillance (ERHMS) https://www.cdc.gov/niosh/erhms/default.html	—
Assessment of Chemical Exposures (ACE) toolkit https://www.atsdr.cdc.gov/ntsip/ace_toolkit.html	—
Chemical hazards: lead information for workers https://www.cdc.gov/niosh/topics/lead/safe.html	Instituto Nacional para la Seguridad y Salud Ocupacional (NIOSH) plomo https://www.cdc.gov/spanish/niosh/topics/plomo.html
Chemical hazards: resources for emergency responders for chemical or radioactive materials https://www.cdc.gov/niosh/topics/emres/chemagent.html https://www.atsdr.cdc.gov/substances/ToxEmergency.asp	Seguridad de productos químicos https://www.cdc.gov/spanish/niosh/topics/quimicos.html
Preventing carbon monoxide poisoning from small gasoline-powered engines and tools https://www.cdc.gov/niosh/docs/96-118/	Prevención de envenenamiento con monóxido de carbono producido por herramientas y equipos con motores pequeños de gasoline https://www.cdc.gov/spanish/niosh/docs/96-118_sp/
Heat and outdoor workers https://www.cdc.gov/disasters/extremeheat/workers.html	Los trabajadores al aire libre y el calor https://www.cdc.gov/extremeheat/espanol/workers_esp.html
Indoor environmental quality https://www.cdc.gov/niosh/topics/indoorenv/	—
Indoor environmental quality: preventing occupational respiratory disease from exposures caused by dampness in office buildings, schools, and other nonindustrial buildings https://www.cdc.gov/niosh/docs/2013-102/	Prevención de enfermedades respiratorias ocupacionales por exposición causadas por la humedad en edificios de oficinas, escuelas y otros edificios no industriales https://www.cdc.gov/spanish/niosh/docs/2013-102_sp/
Indoor environmental quality: recommendations for the cleaning and remediation of flood-contaminated HVAC systems: a guide for building owners and managers https://www.cdc.gov/niosh/topics/emres/Cleaning-Flood-HVAC.html	—
Safety: guidance on personal protective equipment and clothing for flood cleanup workers https://www.cdc.gov/niosh/topics/emres/ppe-flood.html	Equipo de protección personal y la ropa para las personas que trabajan en la limpieza después de las inundaciones https://www.cdc.gov/spanish/niosh/topics/flood_sp/ppe-flood_sp.html
Safety: information for response and cleanup workers https://www.cdc.gov/disasters/hurricanes/workers.html	Información de seguridad para trabajadores de respuesta a emergencias y de limpieza https://www.cdc.gov/es/disasters/hurricanes/workers.html
Worker safety after a flood https://www.cdc.gov/disasters/floods/workersafety.html	Seguridad de los trabajadores después de una inundación https://www.cdc.gov/es/disasters/floods/workersafety.html
Traumatic incident stress: symptoms and recommendations for responders https://www.cdc.gov/niosh/topics/traumaticincident/	Estrés por sucesos traumáticos: Información para el personal de emergencia https://www.cdc.gov/spanish/niosh/docs/2002-107_sp/
Tree removal: preventing chain saw injuries during tree removal after a disaster https://www.cdc.gov/disasters/chainsaws.html	Cómo prevenir lesiones causadas por motosierras después de un desastre https://www.cdc.gov/es/disasters/psa/chainsaw.html
Tree removal: preventing falls and electrocutions during tree trimming https://www.cdc.gov/niosh/docs/92-106/	Retiro de árbol: prevención de caídas y electrocuciones durante la poda de árboles https://www.cdc.gov/spanish/niosh/docs/92-106_sp/

* Information on this webpage is available in 11 different languages.

^†^ Currently not available in Spanish.

^§^ This fact sheet is available in six additional languages, available at https://www.cdc.gov/co/factsheets.htm.

Hurricane Harvey made landfall on the Texas coast on August 25, 2017, as a Category 4 storm. In southeast Texas, record rainfall caused extensive flooding and damage to public infrastructure and communities, and displaced thousands of persons. As of September 12, 2017, the media have reported >80 storm-related deaths attributed to Hurricane Harvey (medical examiner confirmation is pending for some deaths). Most of these deaths likely were caused by drowning in flood waters within the first few days after impact (e.g., drowning at home or in vehicles).

On September 7, 2017, a Category 5 hurricane, Irma, reached the Lesser Antilles, including the U.S. territories of Puerto Rico and the Virgin Islands. Hurricane Irma then continued its path across the Greater Antilles and made landfall in south Florida on September 10, 2017. Irma’s hurricane-force winds and related storm surges caused substantial damage in the Caribbean and Florida.

Many areas in Texas, Louisiana, Florida, Georgia, and the U.S. territories affected by these storms are still experiencing disruptions in essential services, including electricity, potable water, food, and communications. Numerous health care and public health systems sustained damage. Environmental health impacts from the hurricanes included effects on industries, chemical plants, and hazardous waste sites. Many displaced persons remain in shelters or other temporary housing.

As part of the overall U.S. Department of Health and Human Services response and recovery operations, CDC and ATSDR are supporting public health and medical care functions for affected communities and persons displaced by the hurricanes. As of September 12, 2017, CDC and ATSDR had sent pharmacy and federal medical station supplies to Texas, Louisiana, and Florida. CDC and ATSDR have also activated and deployed members of the U.S. Public Health Service Commissioned Corps and other personnel to provide technical support for critical public health functions. Field operations and the CDC and ATSDR Emergency Operations Center are supporting mortality and morbidity surveillance; public health messaging and risk communication; water, sanitation, safety, and facility assessments; community rapid needs assessments; mold abatement; industrial and residential contaminant exposure prevention; and vector control.

There are potential public health and safety concerns after hurricane impact. Many injuries and illnesses from hurricanes and floods occur during the response and recovery phases. Common hazards include vehicle- and nonvehicle-related drowning, carbon monoxide poisoning (e.g., from any gasoline-powered engine, including generators and clean-up equipment), electrocution, falls, lacerations, and exposure to mold and industrial and household chemicals (*1*–*8*). In addition, exacerbation of existing chronic conditions and development of acute mental health symptoms are frequent reasons for seeking health care services following a disaster (*9*–*11*). Guidance and other resources to assist in addressing many of these hazards and risk are available (Table 2).

CDC and ATSDR also offer a disaster response clinical consultation service to assist health care providers, public health professionals, and emergency response partners. This service can be accessed by emailing CDC IMS Clinical Inquiries at eocevent168@cdc.gov.

For additional assistance, health care providers, public health professionals, and members of the public can also use CDC and ATSDR’s information service, CDC-INFO. Live agents provide up-to-date science-based health information. CDC-INFO can be reached Monday through Friday from 8:00 a.m. to 8:00 p.m. Eastern Time at 1–800-CDC-INFO (1–800–232–4636) or by submitting a web-based form (https://wwwn.cdc.gov/dcs/ContactUs/Form). Services are available in English and Spanish.
